# A Case Study of Improving Yield Prediction and Sulfur Deficiency Detection Using Optical Sensors and Relationship of Historical Potato Yield with Weather Data in Maine

**DOI:** 10.3390/s17051095

**Published:** 2017-05-11

**Authors:** Lakesh K. Sharma, Sukhwinder K. Bali, James D. Dwyer, Andrew B. Plant, Arnab Bhowmik

**Affiliations:** 1 Department of Cooperative Extension, University of Maine, 57 Houlton Rd, Presque Isle, ME 04769, USA; jimdwyer@maine.edu; 2 Maine Potato Board, 744 Main Street, Suite 1, Presque Isle, ME 04769, USA; aplant@mainepotatoes.com; 3 Department of Ecosystem Science and Management, 248 Ag Sciences and Industrial Building, Pennsylvania State University, University Park, PA 16802, USA; axb594@psu.edu

**Keywords:** NDVI, sensor, nitrogen, sulfur, leaf area index and weather

## Abstract

In Maine, potato yield is consistent, 38 t·ha^−1^, for last 10 years except 2016 (44 t·ha^−1^) which confirms that increasing the yield and quality of potatoes with current fertilization practices is difficult; hence, new or improvised agronomic methods are needed to meet with producers and industry requirements. Normalized difference vegetative index (NDVI) sensors have shown promise in regulating N as an in season application; however, using late N may stretch out the maturation stage. The purpose of the research was to test Trimble GreenSeeker^®^ (TGS) and Holland Scientific Crop Circle™ ACS-430 (HCCACS-430) wavebands to predict potato yield, before the second hilling (6–8 leaf stage). Ammonium sulfate, S containing N fertilizer, is not advised to be applied on acidic soils but accounts for 60–70% fertilizer in Maine’s acidic soils; therefore, sensors are used on sulfur deficient site to produce sensor-bound S application guidelines before recommending non-S-bearing N sources. Two study sites investigated for this research include an S deficient site and a regular spot with two kinds of soils. Six N treatments, with both calcium ammonium nitrate and ammonium nitrate, under a randomized complete block design with four replications, were applied at planting. NDVI readings from both sensors were obtained at V8 leaf stages (8 leaf per plant) before the second hilling. Both sensors predict N and S deficiencies with a strong interaction with an average coefficient of correlation (*r*^2^) ~45. However, HCCACS-430 was observed to be more virtuous than TGS. The correlation between NDVI (from both sensors) and the potato yield improved using proprietor-proxy leaf area index (PPLAI) from HCCACS-430, e.g., *r*^2^ value of TGS at Easton site improve from 48 to 60. Weather data affected marketable potato yield (MPY) significantly from south to north in Maine, especially precipitation variations that could be employed in the N recommendations at planting and in season application. This case study addresses a substantial need to revise potato N recommendations at planting and develop possible in season N recommendation using ground based active optical (GBAO) sensors.

## 1. Introduction

**Figure 1:**
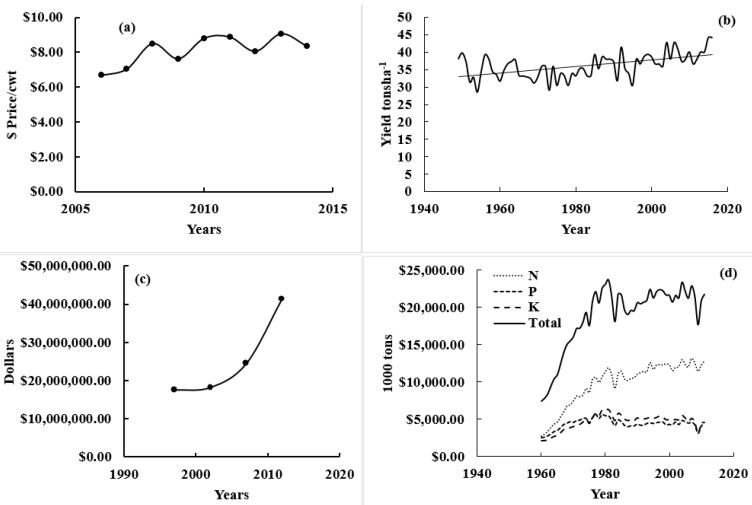
Potato price volatility in the United States over the years (**a**); potato yield volitility in the United States over the years (**b**); input (fertilizer totals, including lime and soil conditioners, measured in $ (USD) expense increased over time in Maine (**c**); and fertilizer use for potatoes in the United States over the years (**d**). Source: USDA, National Agricultural Statistics Service, and New England Ag Statistics.

Management of nitrogen (N) in potatoes is a well-established challenge not entirely owing to yield dependence but also because of quality. Increasing input cost on fertilizers (Figure 1) has made it impractical for producers to gain a competent revenue with a persistent potato price (Figure 1), which is further indicated by the decreasing number of farms in Maine [1]. Use of three major macronutrients, N, phosphorous (P), and potassium (K), has grown in the United States over the years (Figure 1) placing an ominous threat to the ecosystem. Therefore, improvement in fertilizer use efficiency is needed, especially N because of its high price, use and diminishing abilities.

Maine’s potato business has a vital influence on the state’s market, with a $540 million annual impact, a personal income of more than $233 million, state and local taxes of ~$32 million, and a workforce growth of ~6100 jobs [2]. Despite nearly stable potato yield during the past 20 years, farmer’s profits have declined significantly [3] because of enhanced input cost. Growers can adopt variable N rate management methods which may increase yield and reduce input cost, but there is a need to establish guidelines by understanding N behavior under changing climate and soil conditions.

**Figure 2:**
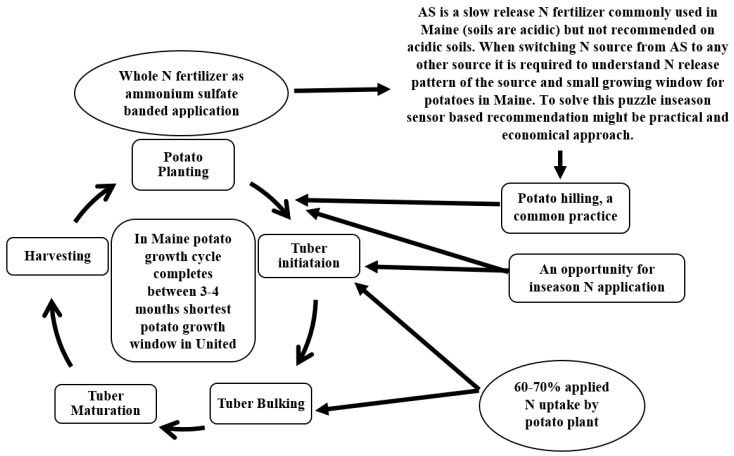
A representation of potato growth cycle in Maine, which lasts ~4 months. Potato hilling is a common agronomy practice that could help in applying in season N.

Nitrogen use efficiency (NUE) for world grain production is low, approximately 33% [4] and it is lower than 50% in potatoes [5]. Low NUE of day-to-day N management methods is owing to the poor synchrony between the N application and crop use (Figure 2) [4,6]. Management of nitrogen in the soil is a complicated process because it requires estimation of a crop’s specific N needs, mineralizable N, and N added to and taken from the soil. Nitrogen management involves managing all potential sources of N over the period of the crop’s lifespan, during high crop demand. The principal cause of N loss is through nitrate leaching or denitrification from extreme precipitation [7,8]*.* Large pre-plant N applications cause poor synchrony between N application and plant N uptake. There is a high use of N in potatoes at the tuber initiation and bulking stage, but 100% of N (average in Maine is 180 kg·ha^−1^) is employed at planting (Figure 2) [9]. This makes N likely to leach in regions of excessive rainfall after crop planting [10], which is common Maine. When applied at planting, N loss reported is between 30% and 60% [11] in corn but might be up to 70% [12]. Therefore, large pre-plant N applications might lead to high levels of available N in the soil profile before actual active plant uptake. Therefore, a farmer requires an understanding of all potential sources of N, including soil N and applied N [13]. In season N application helps to improve NUE compared to pre-plant N application [14,15]. Another component that leads to low NUE is spatial and temporal variability within the landscapes. Several investigations have proven economical and environmental benefits for spatially variable N applications [16,17,18,19,20,21]. Soil N supplies, crop N uptake, and N responses differ spatially within fields [22]. Large quantities of N applied as pre-plant into the soil at a uniform rate is at risk of environmental loss in areas of over-application or soils at risk for loss. Another reason for low NUE is outdated N recommendations that encourage over-application of N.

Estimation of crop biomass is used for N rate measurement with C4 plants [23]. Predicting crop yield is virtually impossible owing to yearly fluctuation in rainfall and temperature, especially in dryland agriculture. In the past, several means were investigated to improve assessment of target yield. Yields were proportioned over ~5 years to obtain a standard yield, and then employed to determine N rate applications. While it reflects past yield, it is not a satisfactory predictor of prospective yields. The N recommendation determined by averaged previous yield found weakly correlated with the economic optimum N rate (EONR) [24] in wheat and corn. Thus, the yield expectation method is not a certain tool for N recommendation. 

Applying optimum N rates allows the potential to improve N use efficiency, profitability, and crop yield with few environmental health hazards [25]; however, N rate application adjustment based on soil and weather situations (Tremblay, 2004) has not been established in potatoes. Several methods are being studied to manage N in potatoes and other commercial crops, e.g., soil and plant analysis [9,10], plant tissue analysis [26], and fertilizer placement and its timing [27]. Likewise, the leaf area index (LAI) has also been utilized for N management. Each has distinct disadvantages such as time-consuming, intense labor utilization, and variable outcomes. Currently, the recommended N rate applied to potatoes in Maine depends on yield predictions and prior year fall soil nitrate test [27]. Crops that typically need N benefit having a leguminous crop follow them in the rotation. The present N recommendations do not use temperature, rainfall, and soil as predictive variables. Climate and soil textures are the major determinants of soil organic matter decomposition [28] and N response [29]. Regional climate, including temperature and precipitation, influence the availability of soil N and the mineralization rate of residues and organic matter [30]. Under wet soil environments, yield and N response improve substantially in coarse textured soil than fine textured soils owing to N volatilization and denitrification under fine textured soils (due to the high water holding capacity of fine textured soils) [31]. 

In addition to N problems in North America, soil sulfur (S) levels are decreasing because of the recent emphasis by the Environmental Protection Agency (EPA) on reducing S emissions from power plants, vehicles, and industries. Growers can easily determine whether EPA regulations on reducing S in the air are working because their crops are showing a sulfur deficiency. The S is needed by crop plants to synthesize certain S-containing amino acids such as cysteine, cystine, and methionine, which are vital components of many proteins. The yield of the crop is a protein-dependent output because protein to obtain maximum economic yield. The yield of the crop is protein-dependent output because protein makes up the plant enzymes, DNA, RNA, and most important chlorophyll, which directly related to the photosynthesis.

Most nutrients, such as phosphorous (P), potassium (K), zinc (Zn), and manganese (Mn), required by the crop plants are applied during early life; however, N and S could be supplied later during in season application. The S deficiency is found to be prevalent on sandy, well drained, and medium textured soils. Deficiency of S is most common in higher landscape positions with thin “A” horizon and coarse-textured soils [32]. N and S have very strong interaction, as observed by Franzen et al. [15]. N and S share common amino acids cysteine, cystine, and methionine, therefore, when N is deficient, it movies from lower to higher leaves and carries S with it, thus showing less or no S deficiency symptoms because S is immobile in plants. In contrast, when N is sufficient, no movement of N to the upper, young leaves results in more S deficiency. This results in unique interaction, where S deficiency becomes more severe with high N rates [15]. 

N is usually applied during planting [9]. However, waiting to tuber initiation could help to improve yields by increasing duration of tuber bulking [33]. The weather and soil type also determine the positive impact of the in season N application. The concept of “need basis” fertilization using active optical sensors was proposed by Schepers et al. [34] to reduce environmental nitrate contamination. This technique contributed to maintaining the optimum yield due to increased NUE [35]. 

Leaf spectral properties are also related to leaf morphology and physiology, including the thickness of leaves [36], N content [37], and chlorophyll concentration [38,39]. Plants with deficiency of N, P, K, S, and Mg absorb less light and reflect more light in the visible spectrum (400–700 nm) compared to normal plants [40]. A study on soybean (*Glycine max* L.) showed increased reflection in the visible spectrum 500–600 nm with deficiency of Co, Ni, Zn, As, and P [41,42]. However, red edge range (690–730 nm) shifts to a shorter wavelength [43,44]. After evaluating Fe, S, Mg, and Mn deficiencies in corn, it was determined that leaf chlorophyll concentrations decrease with decreasing micronutrient concentration [45]. Sulfur deficiency resulted in a 50% reduction in chlorophyll *a* concentration in maize [45]. The symptoms of S deficiencies are the symptoms of N deficiencies with varying levels of chlorosis; however, with sulfur deficiency, the symptoms appear first on younger leaves. Within a corn crop N rate study at two coarser textured sites, higher N rates exhibited more yellowing and stunted growth compared to low N rates.

Ground-based active optical (GBAO) sensors were successfully used in wheat, corn, cotton, sunflower, and sugar beet using crop reflectance properties [46,47,48,49,50,51]. Crop reflectance is a ratio of the radiations reflected from an individual leaf or canopy to the incident radiation. Green plants reflectance and transmittance in visible regions of the spectrum (400–700 nm) is small due to high absorbance by photosynthetic pigments [52]. Leaves absorb predominantly blue (~450 nm) and red (~660 nm) wavelengths and reflect green (550 nm) wavelengths. Reflectance and transmittance are usually high in the near-infrared (NIR) region of the spectrum (~400–700 nm) because of slim absorbance by subcellular organelles and pigments as well as significant scattering at mesophyll cell wall interfaces [53,54,55]. Reflectance measurements at these wavelengths provide information on the vegetative biomass about exposed soil.

Although there is sufficient research published to support improved NUE application strategies, the farmer adoption rate of these strategies in the United States dryland potato growing regions remains low. Reasons for this low adoption rate include practical considerations of equipment, technical support [56] and regional needs such as soil and crop diversity [57], advising a more accurate soil, climate, and cultural practices based N recommendations, e.g., North Dakota corn calculator [51]. 

Figure 2 explains how a short growth window could affect N management while applying in season N. The diagram also explains the possible opportunity for in season N application between first and second potato hilling. This study was designed to investigate the sensor role in predicting potato yield under dryland agriculture; the response of N sources and its impact on sensor readings; S deficiency detection by the sensor and its potential guidelines; and potential role of weather in determining yield variability.

The following questions were addressed that had not been answered in the past:

1. Can GBAO sensors predict dryland potato yield?
2. Is there any difference in yield predicting abilities of two sensors under dryland potato cultivation?
3. Do N sources affect sensors prediction models?
4. Does LAI help in improving yield and NDVI relationship in potatoes?
5. Could sensors detect S deficiencies in potatoes?
6. Is there any relationship between weather data and dryland potato yield in Maine conditions?

## 2. Material and Methods

### 2.1. Location Treatments

The two research sites used for this study in 2016 were: Aroostook Research Station (ARF), latitude and longitude as 46.668486, −68.013048, a S-deficient site (Table 1), and a farmer field in Easton (Easton site), latitude and longitude as 46.689560, −67.919864, under two soil types. The ARF was used to determine sensor ability to detect S deficiencies. To measure the influence of soil type, two soil textures, silty loam, fine loamy, isotic, frigid Aquic Haplorthods and gravely loam, fine loamy, isotic, frigid Typic Haplorthods (commonly found in Maine), were used for this study. At Easton Site, experimental area was under two soil textures, gravelly loam, and silt loam. Russet Burbanks, a processing potato variety was used for this experiment with standard planting (10 cm deep) and row spacing (91 cm). On both sites, 6 N treatments, 0, 56, 112, 168, 224, and 280 kg·ha^−1^, for each N fertilizer, ammonium nitrate (AN) and calcium ammonium nitrate (CAN), were applied at planting. Potassium (KCl) and phosphorous (triple super phosphate) applications were implemented as recommended by University of Maine Soil Testing Laboratory. Each location size was 46 m × 46 m with 3.7 m × 9 m subplots leaving buffer within the replications and rest of the field. For this study, a randomized complete block design with four replications was used.

### 2.2. Weather Data and Soil Data

To estimate the impact of weather on potato yield and quality, weather data from Caribou, Houlton, Bangor, and Portland, Maine were used. Total cumulative precipitation and temperature were collected to develop their relationship with potato yield. 

Soil samples were collected before planting from both research sites and analyzed. The ARF site was found to be S deficient. S deficiency is not common in Maine because growers use ammonium sulfate as an N source, and that source contains S but on ARF ammonium sulfate is not common N source, which indicated in the soil test results, as the site was S deficient. 

### 2.3. Ground-Based Active-Optical (GBAO) Sensor Descriptions and Sensing Procedure

The GBAO sensors use electric diodes that generate modular light in pulses in specific wavebands. The Trimble GreenSeeker^®^ (TGS, Trimble Navigation Limited, Sunnyvale, CA, USA) sensor measures incident and reflected light from the plant at 660 ± 15 nm (red) and 770 ± 15 nm (NIR). In the Trimble GreenSeeker^®^, light emits from the diodes in alternate bursts (visible source pulses for 1 ms and then the NIR diode source pulses for 1 ms at 40,000 Hz). Each light burst from a given source sums to ~40 pulsations before it pauses and then the other diode to emit ~40 pulsations (another 40 pulses). The field of view is constant for heights between 60 and 120 cm above the canopy. The illuminated area is ~60 cm wide and ~1 cm long. The long dimension typically positioned perpendicular to the direction of travel. Outputs from the sensor are NDVI the simple ratio (visible/NIR). 

The Holland Scientific Crop Circle™ ACS-430 (HCCACS-430, Holland Scientific, Inc., Lincoln, NE, USA) sensor simultaneously emits three bands; two in the visible range (red 650 nm, red edge 730 nm) and one in the NIR (760 nm). The light source of the HCCACS-430 is a modulated polychromatic LED array. 

The formula for NDVI and red edge NDVI follows:

NDVI=(NIR−Red)/(NIR+Red)

Red Edge NDVI=(NIR−Red Edge)/(NIR+Red Edge)

Both TGS and HCCACS-430 readings were collected at the V8 growth stage (8 leaf stage of potato plant) of the potatoes from both sites. Sensor data were collected only one time, because of the small growth window and delayed maturity with late N application. Sensor readings were accumulated over the top of the potato canopy from the middle row of each plot where harvest was intended. The TGS and HCCACS-430 readings consisted of an average of 60–70 individual readings from each plot. Means within a treatment were determined using in-house programs for TGS and HCCACS-430 raw data developed for Excel [58]. The LAI was estimated using proprietor proxy LAI (PPLAI) program on the basis of NDVI measured by the HCCACS-430 sensor. 

### 2.4. Harvesting

Two ten-foot rows were harvested in September 2016. A two-row potato digger was utilized to remove the potatoes from the selected rows, and then the potatoes were handpicked. Potatoes from each collected bag were then kept on moving belt for grading separately for four different USDA grades to get the marketable potato yield (MPY) data. Specific gravity was measured using water method:

Weight of potatoes in the air/(Weight of potato in the air−Weight of potatoes in the water)

### 2.5. Statistical Analysis 

Regression analyses were conducted on sensor readings and yield with yield as the dependent variable, and NDVI as the independent variable to evaluate the relationship between yields. All potential regression relationships were conducted, such as linear, quadratic, square root, logarithmic, and exponential. However, exponential relationships (Easton site) and polynomial relationships (ARF) were found with a high frequency of describing the relationships compared to other models. Therefore, exponential and polynomial models were presented throughout this article. SAS GLM was used to compare the N and NDVI treatments. The NDVI was multiplied to PPLAI to determine the impact over NDVI and MPY relationship. The *p*-value of 0.05 probability was used to differentiate the treatments from each other regarding statistical differences between treatments. 

## 3. Results

**Figure 3:**
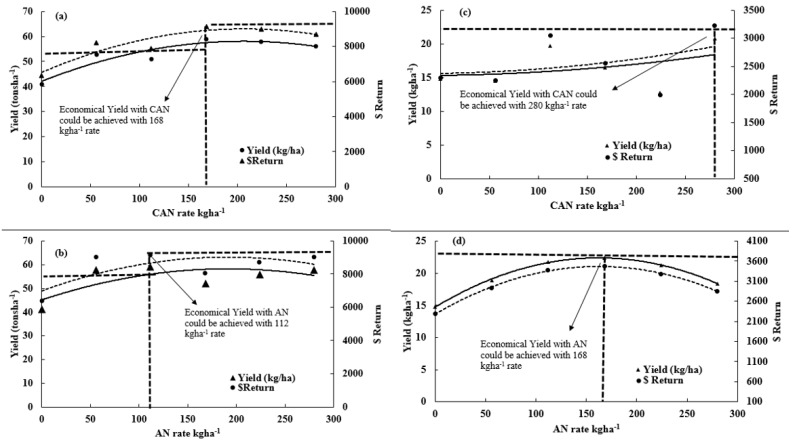
Representing MPY (marketable potato yield) response with CAN (calcium ammonium nitrate) and AN (ammonium nitrate) rates. On the secondary axis, dollar return represents the economic output with increasing yield. (**a**,**b**) Easton site; and (**c**,**d**) ARF site.

**Figure 4:**
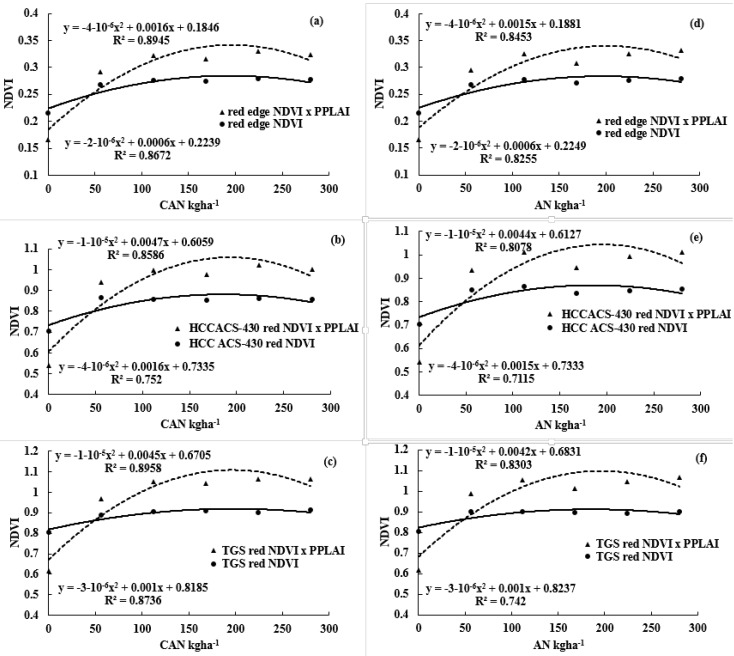
Comparison of the relationship between NDVI (normalized difference vegetative index) and NDVIPPLAI [normalized difference vegetative index multiplied with proprietor proxy leaf area index (PPLAI)] with N sources [CAN (calcium ammonium nitrate) and AN (ammonium nitrate)] and rates [horizontal axis (x-axis)] at Easton site. It is also representing the quadratic curve using NDVIPPLAI with N rates. The relationship between wavelength and N rate was stronger in all the wavelengths and N sources when NDVI multiplied with PPLAI. (**a**–**c**) represents, red edge wavelength (HCCACS-430), red wavelength (HCCACS-430), and red wavelength (TGS) relationship with CAN, respectively. (**d**–**f**) represents, red edge wavelength (HCCACS-430), red wavelength (HCCACS-430), and red wavelength (TGS) relationship with AN, respectively.

**Figure 5:**
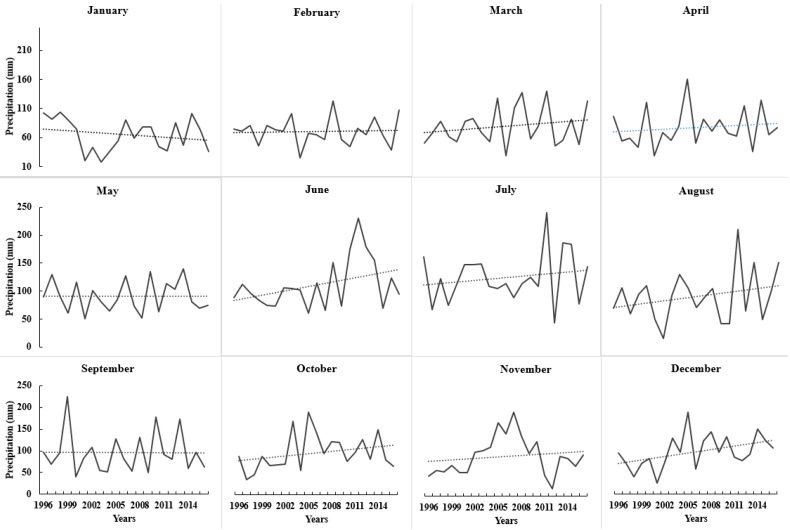
Average monthly precipitation from 1996 to 2016 from January to December. Source: National Weather Service—Gray, Maine.

**Figure 6:**
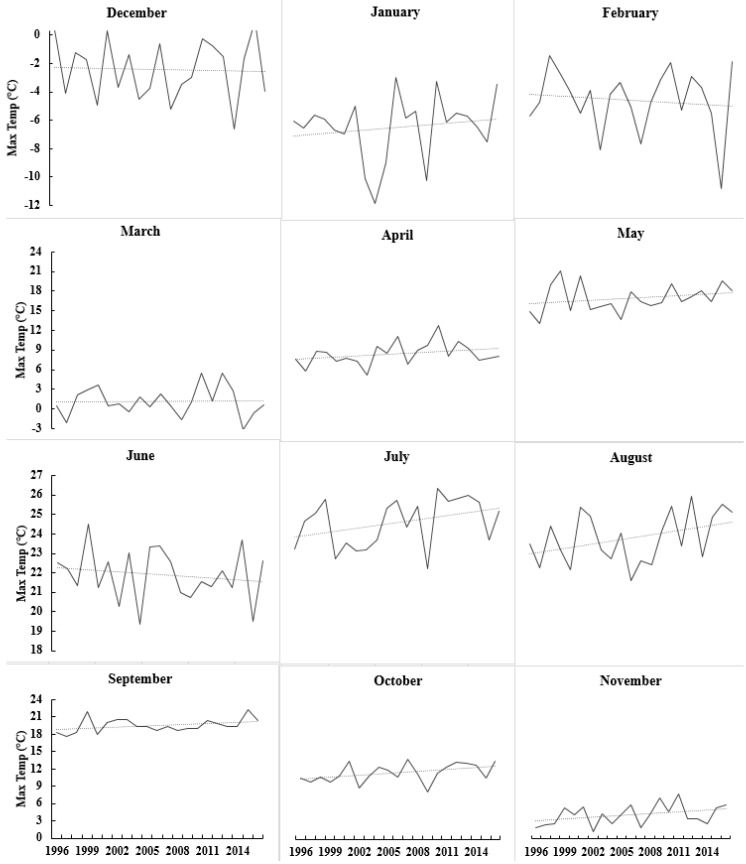
Average maximum monthly temperature variation since 1996 to 2016 from January to December. Source: National Weather Service—Gray, Maine.

**Figure 7:**
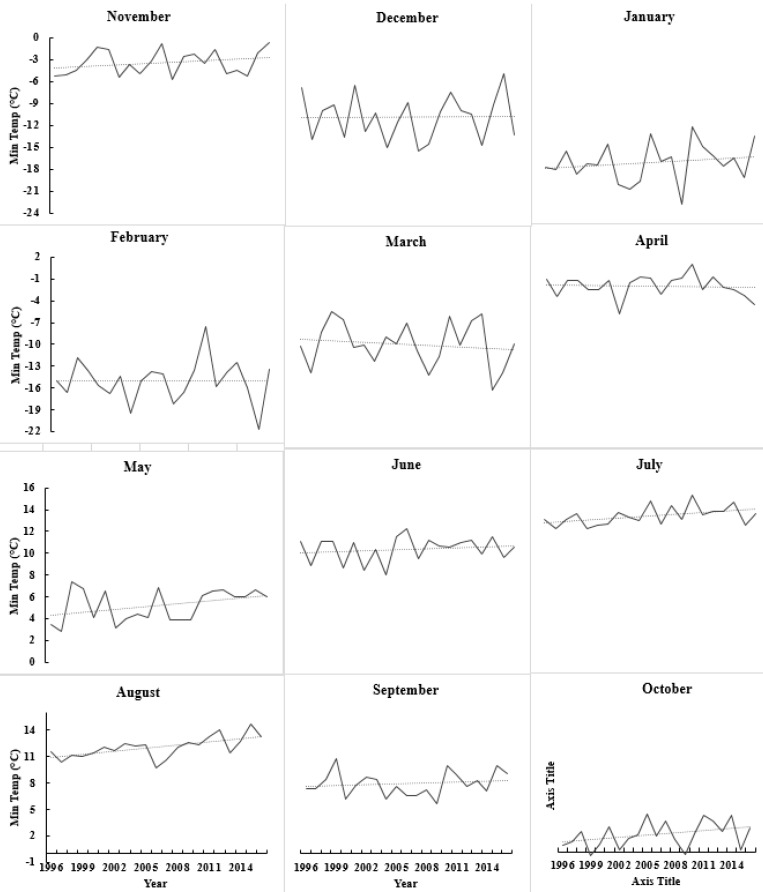
Average minimum monthly temperature variation since 1996 to 2016 from January to December. Source: National Weather Service—Gray, Maine.

**Figure 8:**
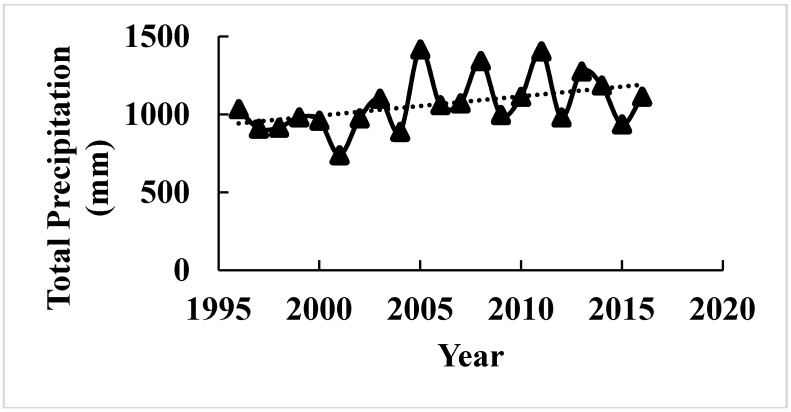
Average yearly precipitation variation since 1996 to 2016 in Northern Maine. Source: National Weather Service–Gray, Maine.

**Figure 9:**
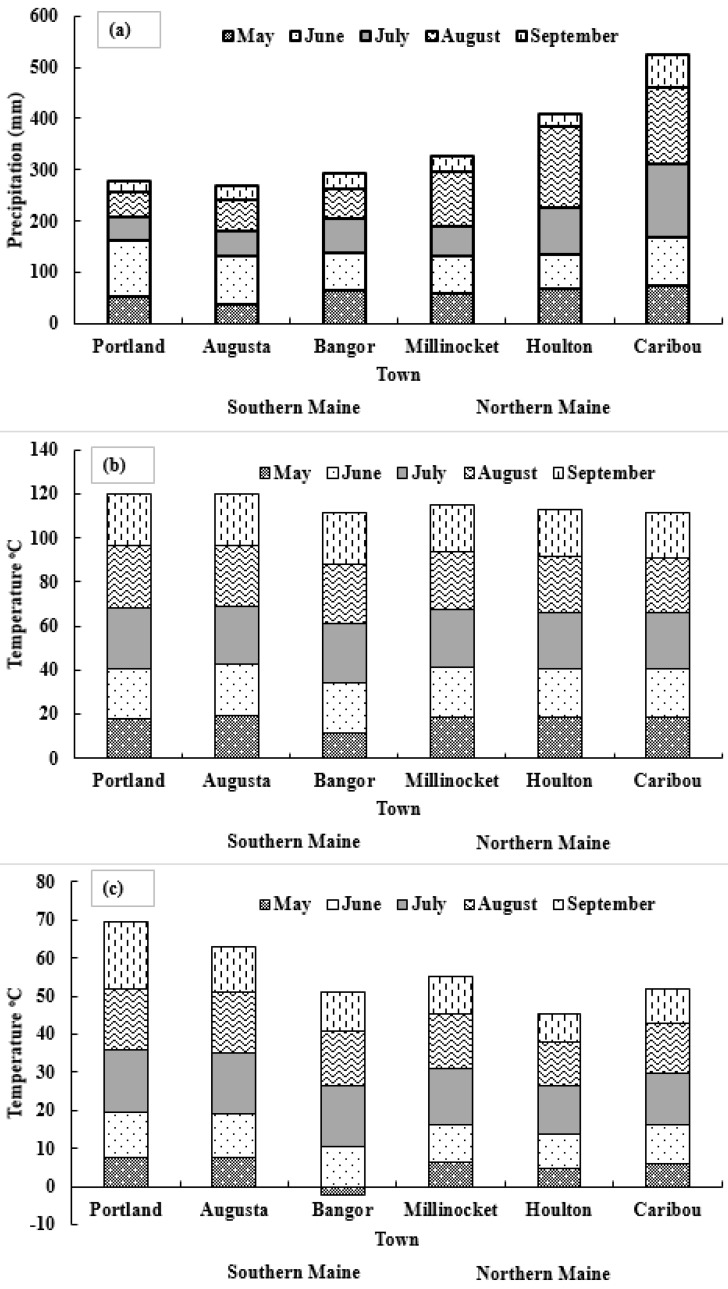
Average monthly precipitation, and maximum and minimum temperature variation from south to north of Maine in 2016. (**a**–**c**) represents average monthly precipitation, and maximum and minimum temperature variation, respectively, over four months (typical growth window for crops in Maine) from south to north of Maine. Source: National Weather Service—Gray, Maine.

Since both sites had specific objectives, they were analyzed separately. The UMaine Soil Test results suggested that there was severe S deficiency, 6 and 10 ppm at 0–15 and 15–46 cm soil depth, at Aroostook Research Farm site (Table 1). Comparison between MPY due to two N sources, the difference in sensors prediction model, the difference in red and red edge wavebands, specific gravity, use of sensors based PPLAI, and weather data were analyzed to address the objectives (Table 2 and Table 3 and Figure 3, Figure 4, Figure 5, Figure 6, Figure 7, Figure 8 and Figure 9).

### 3.1. Nitrogen Analysis, Economics, and Specific Gravity

Variations in N response were found with both CAN and AN at the Easton site. At the Easton site, both N sources behaved similarly towards N response (Figure 3). There was no significant difference found between the yields using two different N sources (Figure 3). However, both AN and CAN produced more yield compared to the control (Figure 3). The yield decreased to 56 t·ha^−1^ with highest N treatment (280 kg·ha^−1^) compared to 59 t·ha^−1^ with 168 and 112 kg·ha^−1^ N with CAN and AN, respectively. Maximum marketable potatoes yield (MPY), 59 t·ha^−1^, were observed with CAN using 168 kg·ha^−1^ and AN using 112 kg·ha^−1^ and minimum yield, 41 t·ha^−1^, was recorded with control (Figure 3). When comparing CAN individually, maximum MPY was observed with 168 kg·ha^−1^ of AN. However, almost similar MPY was found with 112 kg·ha^−1^ of AN (Figure 3). Minimum MPY was recorded with both N sources under control treatment (Figure 3). 

There were variations in economic benefit observed at Easton site (Figure 3). When compared economically, AN was more economical than CAN. The marginal value product (MVP) was found in negative values after 168 kg·ha^−1^ of CAN and 112 kg·ha^−1^ of AN application (Figure 3), which means that, after these rates, the profit will reduce but it again turned to positive values, which implies variations in N response and yield. Potato specific gravity (PSG) was found significantly better with 168 kg·ha^−1^ of AN than any other N treatment. The overall range of PSG observed was between 1.08 (280 kg·ha^−1^ CAN) and 1.09 (168 kg·ha^−1^ AN) (data not presented here). 

At the ARF (S deficient) site, significant variations in N response were observed under both N sources. Maximum MPY, 22 t·ha^−1^, was recorded using 168 kg·ha^−1^ AN (Figure 3). However, minimum MPY, 13 t·ha^−1^, was found with 224 kg·ha^−1^ CAN. At ARF site, AN was found to be better than CAN with regards to MPY (Figure 3). Despite a high N application such as CAN, better MPY was found using the control treatment. When N sources were compared individually, maximum and minimum MPY was recorded with 280 and 224 kg·ha^−1^ of CAN, respectively, which also significantly differed on MVY (Figure 3). Significantly higher MPY, 22 t·ha^−1^, was found with 168 kg·ha^−1^ AN compared to minimum MPY, 15 t·ha^−1^, under control (Figure 3). 

Although ARF was S deficient (6 and 10 ppm at 0–15 and 15–46 cm soil depth), the economic return here could be informative. There was no trend in profitable dollar return observed with CAN, however, with AN, MVP was negative after 224 kg·ha^−1^ rates and the clear trend is found with a sequential increase in AN rates (Figure 3). PSG range at ARF was between 1.07 (112 kg·ha^−1^ CAN) and 1.08 (control). PSG was significantly better under control compared to other treatments (Figure 3). 

When both research sites were compared, Easton was significantly better in MPY with a maximum of 59 t·ha^−1^ compared to ARF, where maximum MPY was 22 t·ha^−1^. The PSG and economics were also better at Easton than ARF. The logic of applying CAN was to see the difference in skin color and toughness due to extra calcium in CAN than AN, but there was no visual difference observed about skin appearance applying CAN over AN.

### 3.2. Sensor Data Analysis

At the Easton site, a high correlation between sensor readings (NDVI) and MPY was found with both the sensors, TGS and HCCACS-430 (Table 2). Considering the small growth window for Maine potatoes, an exponential relationship is used for the best representation of the potato growth curve. However, linear, square root, logarithmic and polynomial relationship were also analyzed, but a weak relationship between MPY and sensor reading was found (data not shown). The models were evaluated using the coefficient of regression (*r*^2^) values for the equation. Comparisons between sensors yield prediction models and wavelengths (red and red edge) were analyzed under both N sources individually and combined. 

Comparing both N sources together, HCCACS-430 was comparatively better than TGS in predicting MPY. Although both the red range wavelength in TGS (*r*^2^ = 0.48) and HCCACS-430 (*r*^2^ = 0.48) had similar red edge wavelength range, the HCCACS-430 (*r*^2^ = 0.57) was significantly better than red range wavelengths (Table 2). The PPLAI relationship with sensor readings (*r*^2^ = 0.58), HCCACS-430, was found to be significant and close to red edge wavelength range. When N sources, CAN and AN, compared individually, TGS red range wavelength performed better (*r*^2^ = 0.59) and resulted in a similar relationship between MPY and NDVI (*r*^2^ = 0.59) as red edge range in HCCACS-430 under CAN application (Table 2). Weaker MPY and NDVI relationship (*r*^2^ = 0.47) was observed with HCCACS-430 red range wavelength. The relationship between PPLAI and NDVI was weakest (*r*^2^ = 0.42) under CAN (Table 2). However, under AN, a high r^2^ value of 0.64 was observed with red range wavelength. Comparing all models under AN, the relationship between MPY and NDVI was weakest (*r*^2^ = 0.60, significant at *p* > 0.005) with red range wavelength from TGS (Table 2). The PPLAI and NDVI relationship (*r*^2^ = 0.64) under AN was found to be close to red range wavelength. When both N sources were compared to predict MPY, AN was consistently better than CAN.

The inverse relationship between NDVI and MPY was found (Table 3) at ARF (S deficient site). Since the location was, S deficient, the site was evaluated to predict S deficiency and to advance S application guidelines. Again, both sensors, TGS and HCCACS-430, wavelengths and N sources were compared. All of the prediction models, linear, square root, exponential, logarithmic, and polynomial, were tested (data not shown), and polynomial found consistently better than all other models. The *r*^2^ value was considered while comparing the model validation. 

When both N sources, CAN and AN, were combined and analyzed, TGS range wavelength was the weakest (*r*^2^ = 0.13) in predicting S deficient MPY compared to *r*^2^ = 0.44 under HCCACS-430 red range (Table 3). Both the wavelength ranges, red edge and red, under HCCACS-430 performed similarly (*r*^2^ = 0.44). The relationship between PPLAI and NDVI was found significant (*r*^2^ = 0.40) (Table 3). When both N sources were analyzed individually, TGS red range wavelength relationship with MPY improved (*r*^2^ = 0.35) but was still found to be weakest among other HCCACS-430 red and red edge range wavelength models, *r*^2^ = 0.44 and *r*^2^ = 0.53, respectively, under CAN application. PPLAI relationship with NDVI was significant (*r*^2^ = 0.50) and better than red range relationship with both the sensors (Table 3). Under AN, no correlation between MPY and NDVI was found using TGS red range. However, a significant relationship observed between MPY and NDVI from red (*r*^2^ = 0.34) and red edge (*r*^2^ = 0.35) wavelength of HCCACS-430. PPLAI and NDVI relationship was significant but weaker than any HCCACS-430 wavelengths (Table 3). When both N sources were compared to predict S deficiency, CAN was comparatively better than AN. There was no relationship observed between sensor readings and PSG at both sites and either N source. 

A consistent weak red range wavelength relationship with MPY was analyzed at the Easton site due to S deficiency found at the ARF site. Figure 4 (scaling adjusted for better visuals) clearly depicts a red edge and red wavelength pattern, with increasing N rate with both CAN and AN. Similar to NDVI, the range is 0–1, red edge range was found between 0 and 0.3. However, red wavelength range was observed between 0 and 0.95, that is ~1. More variations in the absorption spectra were found in red wavelength from HCCACS-430 as compared to a red wavelength from TGS. Wavelength data followed the yield analysis regarding significance that confirmed the strong relationship between MPY and NDVI.

### 3.3. Multiplied PPLAI with NDVI

Using soil moisture to improve NDVI and yield relationship was conducted successfully [59], but it is difficult to measure soil moisture on a large farm (impractical). Moreover, most of the states (weather data) do not provide soil moisture data. An alternative method, multiplying NDVI with PPLAI (calculated by the sensor-HCCACS-430), was used to determine its impact over NDVI and yield relationship. 

A significant improvement in NDVI and yield relationship was observed when NDVI was multiplied with corresponding subplot (treatment plot) PPLAI (Table 2 and Table 3). A significant improvement in the NDVI and MPY relationship was found after multiplying PPLAI with red wavelength NDVI, compared to the red edge NDVI while developing a relationship with MPY under CAN + AN, CAN, and AN at Easton site (Table 2 and Figure 4). A similar trend was observed at the ARF site despite an S deficiency (Table 3 and Figure 4). However, the only relationship was found to be weaker after NDVI, and PPLAI multiplication was at the ARF site using red edge wavelength with CAN application (Figure 4). 

Red and red edge NDVI responses to N rate were analyzed with and without PPLAI multiplication. No significant difference was found using NDVI versus NDVI × PPLAI with N rate from both sensors (TGS and HCCACS-430) (Figure 4). Control red and red edge NDVI and NDVI × PPLAI from HCCACS-430 and TGS was observed to be significantly lower than all other N treatments. Figure 4 shows that NDVI × PPLAI relationship with N rate had a better quadratic curve (relationship) with N level application compared to NDVI alone for all wavelengths. The relationship between N rate and NDVI × PPLAI was found to be stronger than NDVI alone using all wavelengths. 

### 3.4. Weather Data Analysis

Variations in climate occur year to year, and crop productivity depends heavily on its parameters such as precipitation and temperature. Before any recommendation for in season N application, it is important to consider the weather patterns. Weather in northern Maine was found to be extremely variable regarding total monthly precipitation, and maximum and minimum temperature (Figure 5, Figure 6 and Figure 7). The trend of precipitation from March to December (except September) was found to be increasing and significantly variable each month every year starting from 1996 (Figure 5). Total average monthly precipitation for potato growth season (May to August), 98 mm, was found to be similar to the historical average of 102 mm, but average monthly precipitation during July (140 mm) and August (15 cm) was significantly higher than historical averages of 120 and 86 mm, respectively (Figure 5). The average monthly maximum temperature was found to be increasing with time since 1996 during May, July, and August. It was also observed that it was significantly variable with time (Figure 6). However, the average minimum monthly temperature increased during May, August, October, and November since 1996 (Figure 7). Total average yearly maximum and minimum temperatures increased with time and were significantly higher in 2016 (Figure 6 and Figure 7). The average yearly maximum temperature in Aroostook County was 9.92 °C, which was considerably lower than 2016 maximum temperature of 10.93 °C (Figure 6). The average yearly minimum temperature (−0.55 °C) in Aroostook County was significantly lower than the average minimum temperature in 2016 (0.12 °C) (Figure 7). Although total precipitation was found to be increasing since 1996 (Figure 8), it was highly variable within the years, which could persuade drought conditions. For example, in 2016, the average yearly precipitation was found to be higher (1116 mm) than average (1066 mm), but the severe drought was observed in some areas of northern Maine and all southern Maine. The trend of precipitation was analyzed, and clear evidence of low precipitation was found in the south of Maine. A similar pattern in maximum and minimum temperature was found from south to north Maine (Figure 9). 

## 4. Discussion

The variation in N response and economic benefit at the Easton site was analyzed using soil type. A Web Soil Survey was used to locate the soil type, and two types of soils were found (CgB and CoB) under the experimental site as Caribou gravelly loam and Conant silt loam. The variations in N response at the single location might be due to the change in soil types. Similar results were found in North Dakota during a three-year corn fertility study, where soils were divided into different categories depending on their textures [60,61], and soils were categorized as high clay and medium textured soils. They performed multiple regression analysis on all 56 sites. The statistical analysis defines the categories depending upon their N response. However, since we had one site, it was not practical for one site, but our data clearly depicted that N response differed when N passed 150 kg·ha^−1^ with a lower yield but again increased with further increase in N rate. This could be due to the cultivation practices where the soil was mixed from two series by farmer cultivation from several years

Both the research sites were analyzed separately because of the S deficiency at the ARF site. The inverse (decrease in NDVI readings with increasing N rate) relationship between NDVI and NDVIPPLAI with MPY may be due to the strong interaction between N and S, where increasing N application increases S deficiency [15,62]. Similar results were found at two sites (Oakes and Arthur) in North Dakota, where severe S deficiency caused low NDVI readings with increasing N rate in corn at the V6 stage. However, with gypsum application at V6, the S deficiency was corrected, and then high NDVI results were recorded [15]. The INSEY (NDVI/growing degree days) at V6 was 0.000288 and 0.000243 with control and 224 kg·ha^−1^ of N, respectively. However, it improved to 0.000326 in control but 0.000428 with 224 kg·ha^−1^ of N. Clearly, the INSEY improved in high N rate after gypsum application. The light green color was observed with increase in N rate at ARF site. These results again align with North Dakota corn N and S study. Increasing S deficiency with an increase in N rate might be due to limited S movement from older leaves to younger leaves [63]. In control N treatment, N moves from lower to higher leaves and takes S with it because N and S share amino acids, i.e., cystine, cysteine, and methionine. On the other hand, in plants with sufficient N, there is no N movement. Therefore, low S movement from lower to higher leaves results in more S deficiency with higher N rates [14,15].

No visual difference in potatoes skin was observed due to CAN application compared to AN. Similar results were recorded in Sharma et al. [61] where the skin was compared using standardized Munsell soil color book. This might be due to common lime application practice in Maine. The pH range in Maine is low, 5–6 [9], due to historical scab issues in potatoes but with scab resistant variety “Russet Burbank” farmers started increasing their farm soil pH, which resulted in a high lime application thereby adding calcium to the soil. Besides, use of ammonium sulfate is another cause of continuous lime application as it is more acidic than other N fertilizers [9]. 

Sensors could predict potatoes yield early in the growth season that would potentially help farmers to improve their yield potential. The results align with the wheat and corn, sugarbeet, and sunflower study in Oklahoma and North Dakota [48,49,51,57,64]. Red edge was significantly better than a red wavelength from both the sensors. This could be due to saturation effect of red wavelength under high biomass. Similar results were reported in North Dakota with sunflower and corn study [51,57,65] where red wavelength was saturated at high N rates and reached to ~1 NDVI value. Although the sensor predicted MPY better with AN than CAN, no evidence of crop yield difference was found in the literature comparing AN and CAN. Therefore, more studies are needed to confirm the difference in CAN and AN towards yield in Maine, under acidic soils.

Additionally, green leaf reflectance was about 20% in the 500–700 nm range (green to red wavelength) ,whereas green foliage reflection in the red edge range approached 60% in the 700–750 nm range. The red spectrum measured plant biomass, but it is sensitive to low chlorophyll content (3–5 μg·cm^−2^) [66]. The red edge spectrum (700–750 nm) is sensitive to a wide range of chlorophyll (0.3–45 μg·m^−2^) [66]. The red-edge wavelength measurement differs from the red NDVI analysis because the red-edge measures plant chlorophyll content [43]. Therefore, the red wavelength is saturated at higher crop biomass where red wavelength NDVI values vary in a very narrow range; typically, from 0.9 to 0.99. The high NDVI values referred to as saturation.

There are several methods employed to improve the relationship between NDVI and crop yield like multiplying INSEY (a ratio of NDVI/growing degree days) to crop height [14,49], soil moisture to NDVI [59]. Likewise, saturation-adjusted NDVI [67] and combining crop models to NDVI [68] also improved NDVI yield prediction models. However, they failed to provide a commercial scale algorithm for farmers due to inconsistencies in their results. However, LAI has not been used to a level that could provide growers a stable and reliable sensor based in season N application equation. In this study, consistently positive results were found when PPLAI (calculated by the HCCACS-430) was multiplied with NDVI. The consistency in the results might be due to real time PPLAI collection from HCCACS-430 from potato crop. The significant improvement in red NDVI relationship after PPLAI multiplication could be due to the higher sensitivity of PPLAI measurement compared to red wavelength as red NDVI has the tendency of saturation at high crop biomass [57,69,70,71]. 

Weather in northern Maine was found to be changing with time. For example, the precipitation, and maximum and minimum temperature were found to be increasing, which will eventually influence irrigation, fertigation, and dryland fertility recommendation. The N recommendations in potatoes developed ten years ago. However, the yield, quality, weather, and input cost is different today. Likewise, in season N fertility studies in potatoes in Maine lead to late maturation. However, these studies were conducted ten years ago (unpublished data). Common N application recommendation for potatoes from UMaine soil testing lab is 180 kg·ha^−1^ and 40–44 kg·ha^−1^ credit if there is any leguminous crop in the previous year. In our experiment, 112 kg·ha^−1^ N (AN) produced best MPY, which perhaps could vary with multiple sites, but that is what the intention of presenting this research that multiple soil type data could lead to different prospects of N application. However, it is explaining a need to generate a recommendation that can explain soil variability better, and in season sensor recommendations might be a wise choice [46,51,64,72,73,74].

Increased precipitation in July and August could have a severe impact on the yield as it might lead to N loss as denitrification and leaching because the rate of N uptake in Russet Burbank was recorded to be high during July and any deficiency of N may decrease yield significantly [75]. In addition, the precipitation pattern was found to be highly variable where N application at planting might put growers into a risk of high N and MPY loss. Increasing temperatures have a positive impact on MPY. Yield from last two years in Maine was the highest in the history of the state [76]. Increased average maximum and minimum temperature could speed up the crop development [77], which might help in improving MPY, as demonstrated from Figure 1, when other factors are constant. In contrast, increase in maximum, and minimum temperature could induce drought conditions in situations of low precipitation [72,78,79] as with high transpiration rate potatoes will wilt. Consequently, the impact will result in reducing potato yield for example drought conditions in southern and northern Maine in the year 2016 [80] reduced crop productivity. Therefore, adding these parameters to N (boost crop vegetative growth) recommendations could help in dropping adverse impacts of climate variabilities on crop yield. For example, by practicing in season N recommendations, sensors could predict highest potential yield under drought conditions with optimum N application, which might help in reducing crop leaf biomass, thus less transpiration stress. The year 2016 retained MPY average yield number of 2015 only because of high precipitation in Aroostook County countering high temperature resulted in exceptionally high MPY. For example, in research study plot at Easton site, 59 t·ha^−1^ of MPY found when state average was 44 t·ha^−1^. Using soil moisture as a parameter in calculating N rate at planting and in season has been suggested by Walsh et al. [59] but was found to be difficult for growers to use soil moisture data due to unavailable resources. However, precipitation data perhaps could be utilized as a variable, explaining variable soil moisture, replacing soil moisture in the equations. 

## 5. Conclusions

The maximum economic yield in potato was recorded using less N (112 kg·ha^−1^) than recommended by the University of Maine Soil Testing Services (179 kg·ha^−1^). The soil test analysis is a standard resource for growers to determine N recommendations but, since those recommendations came from ARF and significant difference in ARF and farmer site was recorded with respect to S deficiency and N response, there is a need to revise those recommendations. The sensor could predict MPY before second row-hilling and very close to first row-hilling (Figure 2), which created a potential for GBAO sensor to recommend N rate depending on maximum yield potential from that field and variety when there are several commercial potato varieties grown in the area, and each variety has different maximum yield potential. The relationship between MPY and sensor NDVI improved using PPLAI from HCCACS-430, which may help in improving already developed algorithms for wheat, corn, cotton, and sunflower. The relationship between red NDVI and MPY perhaps enhanced even when saturated at higher crop biomass, thus may improve algorithm developed in red NDVI to predict high biomass crop yield such as corn. Sensor predicted S deficiency when N was variable (different rates applied). Still, the sensor could not tell which nutrient is deficient in an on-farm situation when N rates are unknown. However, when regular field sensor readings will be compared to the plot that had more N, there will be clear evidence of S deficiencies because of the inverse relationship due to N and S interactions which will be detected by the sensor algorithm by introducing inverse algorithm option in cases field NDVI is better than high N plot but detecting S deficiency by farmer will further depend on its educational training and nutrient response understanding. Weather data affected MPY significantly from south to north in Maine, especially precipitation variations, which could be employed in the N recommendations at planting and in season application. This case study addresses a substantial need to revise potato N recommendations at planting and develop possible in season N recommendation using GBAO sensors. 

## Figures and Tables

**Table 1:** Comprehensive soil test before planting at Easton and Aroostook Research Farm sites. Soil samples were collected at two depths, 0–15 cm and 15–46 cm, to analyze the soil nutrient levels thoroughly.

Location/Soil Sample Depth	OM	pH	P	K	Ca	Mg	N	S	B	Cu	Fe	Mn	Zn
	%		Kg·ha^−1^	% Saturation			ppm						
**Easton/0–15 cm**	3.4	5.4	39.9	13.5	38.4	13.9	26	133	0.5	1.25	4.9	5.4	1.0
**Easton/15–46 cm**	3.1	5.5	44.2	17.1	40.1	13.6	18	167	0.4	1.19	4.6	6.1	1.0
**ARF/0–15 cm**	4.0	5.4	36.0	3.3	54.4	7.0	24	6.0	0.2	3.27	9.7	4.3	0.6
**ARF/15–46 cm**	2.7	5.3	30.7	2.2	43.5	5.9	12	10	0.2	4.3	15	3.3	0.5

**Table 2:** Regression analysis between MPY ^†^ and NDVI ^‡^ from TGS ^††^ and HCCACS-430 ^‡‡^ at Easton site. The coefficient of determination from the exponential relationship was used to measure the strength of the relationship between MPY and NDVI. Another relationship was developed using sensor PPLAI multiplied with sensor NDVI and then develop a relationship with MPY. The strength of PPLAI ^†‡^ and sensor readings was also determined using regression analysis.

Location	N Source	Sensor Type	Wavelength	NDVI and Yield	(NDVI × LAI) and Yield	NDVI and LAI
Easton	CAN+AN	HCC ACS-430	Red edge	*y* = 13.377e^5.1926*x*^ *R*^2^ = 0.57 ***	*y* = 29.925e^1.9611*x*^ *R*^2^ = 0.58 ***	*y* = 22.741e^0.7791*x*^ *R*^2^ = 0.58 ***
			Red	*y* = 10.941e^1.9033*x*^ *R*^2^ = 0.488 ***	*y* = 28.285e^0.6897*x*^ *R*^2^ = 0.58 ***	
		TGS	Red	*y* = 4.9323e^2.6857*x*^ *R*^2^ = 0.48 ***	*y* = 25.828e^0.7424*x*^ *R*^2^ = 0.60 ***	
	CAN	HCC ACS-430	Red edge	*y* = 243.9*x* − 11.495 *R*^2^ = 0.59 ***	*y* = 95.468*x* + 25.323 *R*^2^ = 0.62 ***	*y* = −1.1685*x* + 83.7 *R*^2^ = 0.42 ***
			Red	*y* = 84.432*x* − 17.199 *R*^2^ = 0.4651 ***	*y* = 32.49*x* + 23.473 *R*^2^ = 0.59 ***	
		TGS	Red	*y* = 5.3235e^2.5815*x*^ *R*^2^ = 0.59 ***	*y* = 26.218e^0.7183*x*^ *R*^2^ = 0.69 ***	
	AN	HCC ACS-430	Red edge	*y* = 257.03*x* − 14.109 *R*^2^ = 0.62 ***	*y* = 99.959*x* + 24.831 *R*^2^ = 0.64 ***	*y* = −1.6606*x* + 99.059 *R*^2^ = 0.64 ***
			Red	*y* = 104.65*x* − 32.411 *R*^2^ = 0.64 ***	*y* = 35.318*x* + 21.984 *R*^2^ = 0.66 ***	
		TGS	Red	*y* = 4.6811e^2.7571*x*^ *R*^2^ = 0.60 ***	*y* = 26.153e^0.7399*x*^ *R*^2^ = 0.70 ***	

^†^ Marketable potato yield; ^‡^ Normalized difference vegetative index; ^††^ Trimble GreenSeeker^®^; ^‡‡^ Holland Crop Circle^®^ ACS-430; ^†‡^ Proprietor proxy leaf area index; *** Denotes significance at 0.001.

**Table 3:** Regression analysis between MPY ^†^ and NDVI ^‡^ from TGS ^††^ and HCCACS-430 ^‡‡^ at ARF site. The coefficient of determination from the polynomial relationship was used to measure the strength of the relationship between MPY and NDVI. Another relationship was developed using sensor PPLAI multiplied with sensor NDVI and then develop a relationship with MPY. The strength of PPLAI ^†‡^ and sensor readings was also determined using regression analysis.

Location	N Source	Sensor Type	Wavelength	NDVI and Yield	(NDVI × LAI) and Yield	NDVI and LAI
ARF	CAN+AN	HCC ACS-430	Red edge	*y* = −93.851*x*^2^ − 65.368*x* + 29.592 *R*^2^ = 0.44 ***	*y* = 263.34*x*^2^ − 133.38*x* + 25.403 *R*^2^ = 0.44 ***	*y* = 3.6912*x*^2^ − 27.691*x* + 29.076 *R*^2^ = 0.44 ***
			Red	*y* = 2.7838*x*^2^ − 21.484*x* + 29.259 *R*^2^ = 0.40 ***	*y* = 14.275*x*^2^ − 30.907*x* + 24.835 *R*^2^ = 0.43 ***	
		TGS	Red	*y* = 136.23*x*^2^ − 228.73*x* + 113.74 *R*^2^ = 0.13 *	*y* = 13.803*x*^2^ − 35.707*x* + 28.8 *R*^2^ = 0.43 ***	
	CAN	HCC ACS-430	Red edge	*y* = 924.38x^2^ − 414.15*x* + 59.023 *R*^2^ = 0.53 ***	*y* = 1039*x*^2^ − 277.72*x* + 31.693 *R*^2^ = 0.50 ***	*y* = 0.1235*x*^2^ − 7.1541*x* + 117.81 *R*^2^ = 0.49 ***
			Red	*y* = 60.696*x*^2^ − 100.34*x* + 55.745 *R*^2^ = 0.44 ***	*y* = 64.08*x*^2^ − 67.932*x* + 31.369 *R*^2^ = 0.48 ***	
		TGS	Red	*y* = 168.64*x*^2^ − 278.12*x* + 130.41 *R*^2^ = 0.35 **	*y* = 59.946*x*^2^ − 76.598*x* + 37.495 *R*^2^ = 0.52 ***	
	AN	HCC ACS-430	Red edge	*y* = 163.38*x*^2^ − 123.64*x* + 32.407 *R*^2^ = 0.35 **	*y* = 487.19*x*^2^ − 152.39*x* + 25.469 *R*^2^ = 0.34 **	*y* = −0.0048*x*^2^ + 0.8875*x* − 6.7679 *R*^2^ = 0.26 **
			Red	*y* = 17.743*x*^2^ − 34.495*x* + 31.499 *R*^2^ = 0.34 **	*y* = 34.634*x*^2^ − 38.558*x* + 25.071 *R*^2^ = 0.34 **	
		TGS	Red	*y* = 153.57*x*^2^ − 253.26*x* + 123.46 *R*^2^ = 0.03	*y* = 7.0663x^2^ − 28.936*x* + 27.383 *R*^2^ = 0.32 **	

^†^ Marketable potato yield; ^‡^ Normalized difference vegetative index; ^††^ Trimble GreenSeeker^®^; ^‡‡^ Holland Crop Circle^®^ ACS-430; ^†‡^ Proprietor proxy leaf area index; *** Denotes significance at 0.001, ** denotes significance at 0.01, and * denotes significance at 0.05.
